# Composition and Neurogenetic Effects of Embryonic Cerebrospinal Fluid: A Systematic Review

**DOI:** 10.1007/s12017-025-08829-1

**Published:** 2025-05-10

**Authors:** Ana Călina Beldean, Radu Cristian Moldovan, Olga Sorițău, Ștefan Strilciuc, Răzvan Ciortea, Fior Dafin Mureșanu, Alina Vasilica Blesneag, Ștefan Florian, Alexandru Cristian Bolunduț, Sergiu Șușman

**Affiliations:** 1 Department of Morpho-Functional Sciences, “Iuliu Haţieganu” University of Medicine and Pharmacy, 400012 Cluj-Napoca, Romania; 2 MedFuture-Research Center for Advanced Medicine, “Iuliu Haţieganu” University of Medicine and Pharmacy, 400337 Cluj-Napoca, Romania; 3 Laboratory of Tumor Cell Biology and Radiobiology, Institute of Oncology “Prof. Dr. Ion Chiricuță”, 400015 Cluj-Napoca, Romania; 4 Research Center for Functional Genomics, Biomedicine and Translational Medicine, “Iuliu Haţieganu” University of Medicine and Pharmacy, 400337 Cluj-Napoca, Romania; 5 Department of Obstetrics and Gynaecology, “Iuliu Haţieganu” University of Medicine and Pharmacy, 400337 Cluj-Napoca, Romania; 6 Department of Neurosciences, “Iuliu Haţieganu” University of Medicine and Pharmacy, 400012 Cluj-Napoca, Romania; 7 Neurology Department, Emergency County Hospital, 400012 Cluj-Napoca, Romania; 8 Department of Neurosurgery, Emergency County Hospital, 400012 Cluj-Napoca, Romania; 9 1st Department of Pediatrics, “Iuliu Haţieganu” University of Medicine and Pharmacy, 400370 Cluj-Napoca, Romania; 10 Department of Pathology-Neuropathology—Imogen Research Center, Emergency County Hospital, 400012 Cluj-Napoca, Romania

**Keywords:** Embryonic cerebrospinal fluid, Systematic review, Composition, Neurogenetic effects

## Abstract

Embryonic cerebrospinal fluid (E-CSF) has an important role in neurological development. Due to limited availability, the composition and properties of E-CSF are not known to the present. Our review aims to offer a comprehensive perspective over the studies published to date regarding the composition and effects of E-CSF. We performed a systematic search of four databases for studies regarding normal E-CSF, according to the Preferred Reporting Items for Systematic Reviews and Meta-Analyses (PRISMA) guidelines. We screened 725 records for eligibility criteria, resulting in 44 studies included in the narrative synthesis. Of these, four compared E-CSF with postnatal CSF, and three studies used human E-CSF for composition description. The most comprehensive set of molecular analyses was performed via mass spectrometry, in four studies. We observed a decrease in the number of published studies in the last 5 years. All included studies showed better results when cells were cultured in E-CSF than basal medium. Research on E-CSF remains sparse, particularly concerning its role in human developmental neurobiology. The heterogeneous nature of the study designs and experimental approaches showcase the need for standardized methodologies to better understand the unique properties and potential clinical applications of E-CSF.

**Supplementary Information:**

The online version contains supplementary material available at 10.1007/s12017-025-08829-1.

## Introduction

International organizations, such as Human Proteome Organization (HUPO) started initiatives to promote the study of the human proteome (Omenn et al., 2023). As part of this project, the proteomics of nervous tissues has been proposed as a research direction within the Human Brain Proteome Project (HBPP) (Meyer et al., 2003). These initiatives have led to a substantial increase in the number of published articles on the cerebral proteome. However, the study of the composition and protein content of cerebrospinal fluid (CSF) has not been approached with the same breadth, with a much lower number of scientific articles published on this subject to date. Nevertheless, an approximate characterization of the human CSF proteome (at least qualitative) has been achieved, with over 3000 identified proteins (Macron et al., 2018).

Due to the limited availability of human fetal tissues, the proteome of fetal and embryonic human CSF has not been characterized. Compared to adult CSF or other fetal tissues, fewer studies that use mass spectrometry or other methods to characterize the proteome of embryonic and fetal human CSF have been conducted to date, offering no comparative perspective with adult human CSF.

In addition to its trophic and physical protection roles, CSF seems to play an important regulatory role, modulating the dynamics of stem cells and tissue proliferation and differentiation. Fetal and embryonic CSF, with a specific composition different from that of adult CSF and dependent on the gestational age, might have specific roles (Fame & Lehtinen, 2020). These effects have been studied using embryonic or fetal CSF of animal origin, as well as adult CSF (animal or human), but, up to the present moment, no studies have used human embryonic or fetal CSF on cell cultures.

The neurogenic niche represents a specialized micromedium that interacts with the neural precursors in a specific way, directing the fate of these cells (Scadden, 2006). There is an interplay between a progenitor’s capacity to proliferate and differentiate into mature cells and the characteristics of the niche, dependence that is present in both adult and fetal developing brain. The mechanisms through which the neurogenic niche exerts its effects are inherent and not completely discovered, showing significant differences between the adult and prenatal brain (Silva-Vargas et al., 2016). Adult neurogenic niches exhibit a reduced capacity for neural regeneration compared to embryonic stages, but recent studies showed a potential activation or augmentation of these niches (Bonafina et al., 2020). Therefore, arises the idea of the possible use of embryonic CSF (E-CSF) as a mimic of the fetal niche in the adult brain. Potentially, E-CSF could reactivate dormant pathways in the adult niche, giving a boost to the regeneration capacity of the adult brain zones dedicated to neuroregeneration, and opening multiple practical applications.

The molecular composition of E-CSF has an important translational potential. Identifying the key E-CSF components that promote neurogenesis during development could eventually lead to the engineering of targeted therapies that can stimulate or regenerate neural tissue in adult brains. Understanding the precise molecular interactions between E-CSF and progenitor cells in adult neurogenic niches is crucial for translating this knowledge into clinical therapies. “Rejuvenating” the damaged neurogenic niches and stimulating the natural repair process would be essential to the development of new therapeutic strategies for neurodegenerative disorders or traumatic brain injury (Chang et al., 2016). As such, the ongoing exploration of E-CSF’s molecular components and effects on neural progenitors not only provides a better understanding of embryonic neurogenesis, but also lays the groundwork for novel, clinically relevant strategies to rejuvenate adult brain plasticity and enhance recovery in neurological diseases.

From our knowledge, there are several literature reviews published to date trying to explain the complex function of prenatal CSF in accordance with the known stages of neurodevelopment (Bueno & Garcia-Fernàndez, 2016; Bueno et al., 2020; Fame & Lehtinen, 2020), but none of them conducted a systematic search. The authors provide insights into understanding the studied pathways, linking data regarding E-CSF to other neurodevelopmental areas such as neural progenitor differentiation, pathological models, or choroid plexus physiology, but there is a need for further experimental studies in this domain.

There are also other reviews (Lehtinen et al., 2011), focusing on the possible effects of E-CSF in the adult brain neurogenic niche, and, based on the few experimental studies published, trying to propose new research areas in this direction, with further clinical implications. These studies support the importance of researching the subject, because the unique properties that E-CSF exerts on progenitor cells during development, could be replicated in the adult neurogenic niche, opening multiple treatment possibilities in various neurological domains.

In order to better identify the limitations of the studies published so far and the gaps in the literature on one hand, and the future directions that are worth pursuing on the other hand, there is a need for a systematic approach to review all literature that focuses specifically on E-CSF. The present review aims to offer a comprehensive perspective over the studies published to date regarding normal embryonic and fetal CSF, divided into two categories: description of the composition and the effects on cell/tissue cultures and in vivo.

## Materials and Methods

A systematic review of the literature on the composition and effects of E-CSF was conducted according to the Preferred Reporting Items for Systematic Reviews and Meta-Analyses (PRISMA) guidelines (PRISMA Checklist in Supplementary Materials). The protocol was registered in the Open Science Framework registry on 30 July 2024 (https://osf.io/u9mx6).

We performed a comprehensive search from 01.01.2000 to 03.06.2024 of 4 **databases:** PubMed, Web Of Science, Embase, and Scopus using the following **search strategy:** (“Cerebrospinal Fluid”[Mesh] AND fetal) OR (“Cerebrospinal Fluid”[Mesh] AND embryonic) OR (“Cerebrospinal Fluid Proteins”[Mesh] AND fetal) OR (“Cerebrospinal Fluid Proteins”[Mesh] AND embryonic) OR (ECSF) OR (“fetal Cerebrospinal Fluid”) OR (“embryonic Cerebrospinal Fluid”)—for PubMed and (ECSF) OR (“fetal Cerebrospinal Fluid”) OR (“embry* Cerebrospinal Fluid”)—for Web Of Science, Embase, and Scopus.

### Study Selection

We applied the following inclusion criteria:

- Studies regarding the chemical composition of fetal or embryonic CSF,
- Studies regarding the in vitro or in vivo effects of fetal or embryonic CSF.

We excluded studies that met our exclusion criteria:

- Systematic reviews, reviews,
- Conference abstracts,
- Studies regarding anatomical modifications,
- Studies regarding specific pathologies.

In order to gain a comprehensive perspective over the subject, we did not exclude studies based on E-CSF species, gestational age or other characteristics. We removed all duplicates and evaluated the quality of the retrieved articles. Two authors independently assessed each title and abstract for inclusion. The selected studies were then analyzed in full text, based on eligibility criteria. Discussion with a third reviewer solved all possible disagreements over the quality of retrieved studies. The authors then reviewed full-text papers and extracted the following data: author, year of publication, title, gestational age of CSF, species CSF, fetus/embryo characteristics (pathologies, genetic, etc.), experiment type, in vivo/in vitro (type of cell culture), comparator, method for substance identification, method for effect evaluation, other objectives, results—identified substances, results—effect on cell cultures/subjects. If the studies did not report data regarding one of the variables, we specified: data not assessed. For quality assessment, we used a modified version of Nature Checklist (Cramond et al., 2016) for fundamental research.

Based on the identified objectives, we included the collected data from each study in the established categories: substance identification and effect in vitro/in vivo. The extracted data were summarized and included in a table reviewed independently by each investigator. The studies were divided into subgroups based on the investigated areas of interest.

## Results—Study Selection

**Fig. 1:**
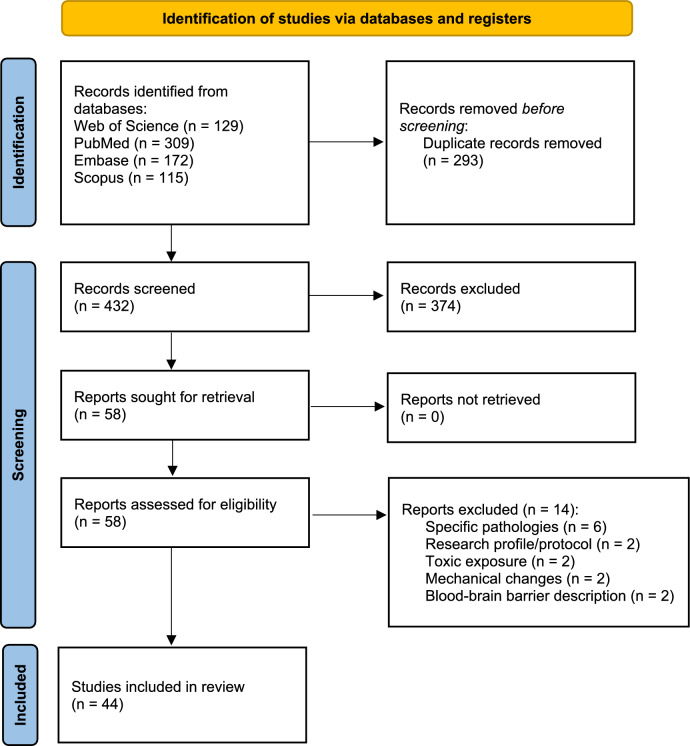
Flow chart of the search results and study selection

After applying the described search strategy, we identified 725 records, from which, after duplicate removal and eligibility assessment, we introduced 44 studies in the analysis (Fig. 1). Outcomes of study quality assessment are summarized in a table (Supplementary Materials). Only 17 of the included studies met at least 50% of the applicable criteria in Nature Checklist.

**Fig. 2:**
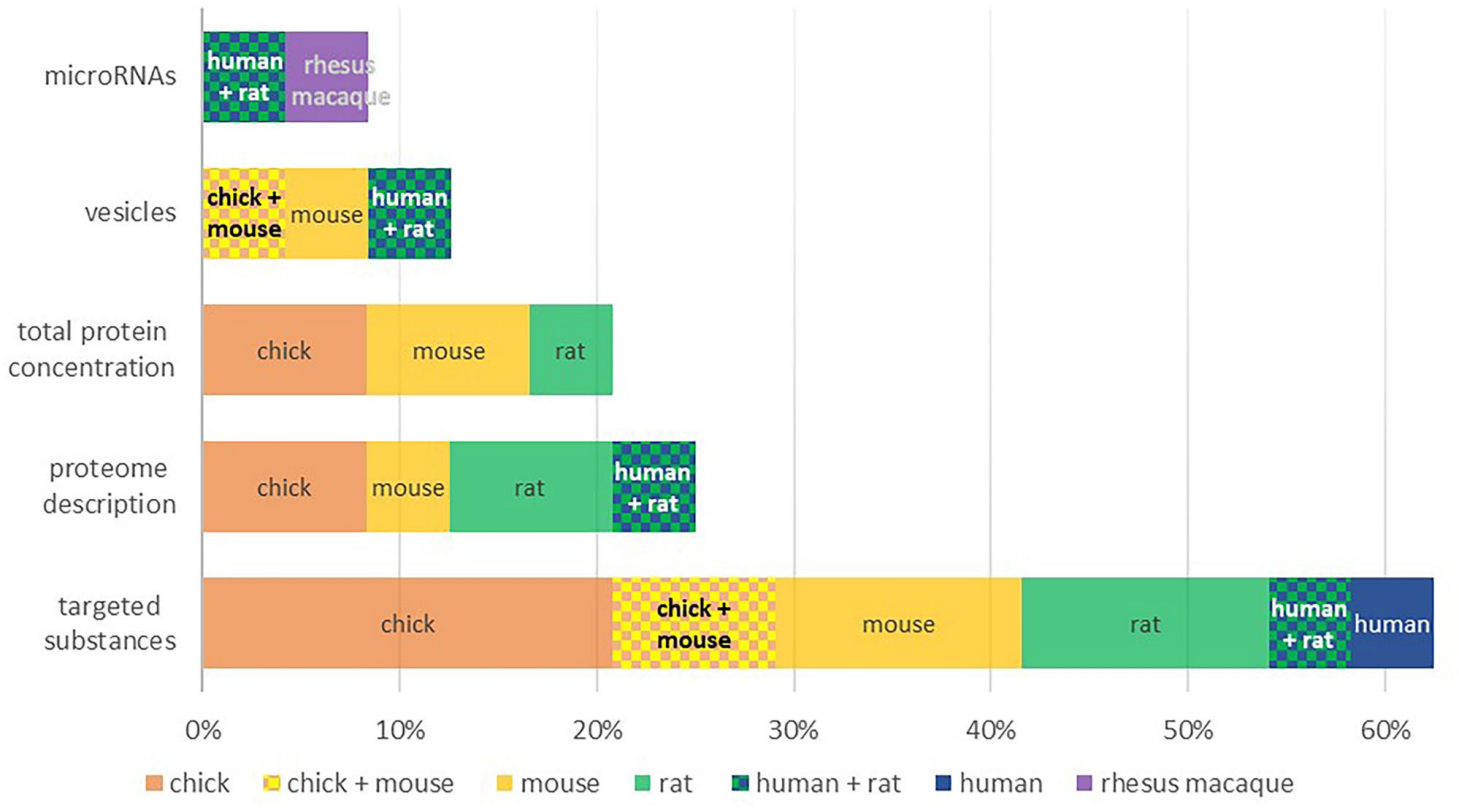
Proportion of studies with different objectives for each species from the total number of studies regarding substance identification (Some studies had multiple types of objectives, therefore they were reported in multiple categories)

**Fig. 3:**
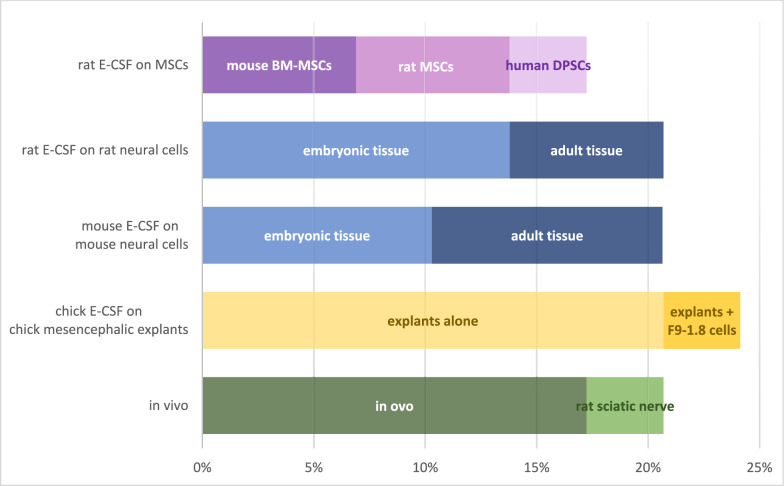
Proportion of studies using each species and each type of experiment from the total number of studies regarding the effect of embryonic cerebrospinal fluid (One study had multiple types of experiments, therefore it was reported in multiple categories. BM-MSCs, bone-marrow mesenchymal stem cells; DPSCs, dental pulp stem cells; E-CSF, embryonic cerebrospinal fluid; MSCs, mesenchymal stem cells)

In the included studies, the CSF was collected from different species. Of the 44 studies, nine focused on both composition and effect, so we chose to describe them in both sections. Consequently, we included in the analysis 24 studies that had as one of the objectives the identification or the measurement of concentration for different substances in the normal embryonic CSF (Fig. 2), the extracted data being summarized in Table 1, and 29 studies that had as one of the objectives the investigation of the effect of normal embryonic CSF on cell cultures or in vivo (Fig. 3), the extracted data being summarized in Tables 2, 3, 4.

**Table 1:** Description of studies regarding substance identification and concentration measurement in embryonic cerebrospinal fluid

Author /year	Species	GA of CSF	Groups and comparator	Method	Results
Christiansen et al. (2000)	Human	17–23 weeks	Different GA	Rocket immunoelectrophoresis in 1% agarose	AFP, albumin, AFP index (constant with GA), CSF-serum ratios (decrease with GA)
Zappaterra et al. (2007)	Human and Rat	Rats: E12.5–17.5 Human: CS20	Rat E-CSF (different GA) vs human E-CSF	One-dimensional in gel electrophoresis and mass spectrometry	188 proteins in human samples 137 proteins common to all rat samples (different GA) 83 proteins common human and rat
				Subgroup analysis	-subcellular localization = similar between rat and human: the majority—secreted proteins, the second—cell membrane proteins -molecular function = similar between rat and human samples
Feliciano et al. (2014)	Human and Rat	Rats: E14-15, Human: 12–18 weeks	Rat vs human E-CSF	1a. Electronic microscopy (rat) + immunoblot (rat + human)	Exosomes present in rat and human E-CSF
				1b. Pathscan analysis of proteins from human and rats E-CSF nanovesicles + Bioinformatics	IGF pathway elements present in exosomes: (p)-STAT3, pAKT, pERK1/2, pSTAT1, pS6, pmTOR, pS6K1 at different relative levels in human E-CSF vs rat E-CSF
				2a. microRNA microarray from rat and human E-CSF nanovesicles + RT-PCR	147 microRNAs in rats (> fourfold enriched) 167 microRNAs in human (16-fold enriched) Confirmation (RT-PCR): evaluation of top 9 microRNAs from rat E-CSF
				2b. Ingenuity Pathway Analysis + Bioinformatics	15 microRNAs significantly different expressed in human vs rat IGF/Insulin-activated microRNA node in both rat and human E-CSF
Parada et al., (2005a, 2005b)	Rat	E12.5	Rat E-CSF vs chick E-CSF (HH24)	2-DE and MS (ESI–MS/MS)	31 proteins in rat E-CSF in 7 groups (enzyme and enzyme regulators only in rats, more apolipoproteins family in rats than chicks)
Simamura et al. (2010)	Rat	E13.5–17.5	Different GA E-CSF vs fetal serum	ELISA	LIF levels (Fetal serum, E-CSF) peaked at E15.5, then decreased at E17.5
Nabiuni et al. (2012)	Rat	E17-20	Different GA	Bradford method	Protein concentration progressively decreases from E17 to E20
Lamus et al. (2020)	Rat	E13.5	NA	Western Blot	FGF2 and EGF
Requena-Jimenez et al. (2021)	Rat	E17-E20	Different GA, normal vs HTx	LC–MS—proteome assessment	E17-18: 849 proteins, E18-19: 982 proteins, E19-20: 730 proteins
				Qiagen ingenuity pathway analysis (IPA)—pathway assessment	xenobiotic related (most significant) HIFalfa1, folate transformations and polyglutamylation, superpathway of serine and glycine, cell cycle control of chromosomal activation, p53
Takata et al. (2022)	Rat	E15.5–19.5	Different GA	ELISA	IGF-1 and IGF-2 levels peaked at E17.5
Ryan et al. (2024)	Rhesus macaque	E155	Normal vs THC (tetrahydro-cannabinol) exposed	qPCR for microRNAs	Normal E-CSF: 77 microRNAs
Gato et al. (2004)	Chick	HH18-30	Different GA and serum proteins	Bradford method	total protein concentration increases with GA
				SDS-PAGE and silver staining	21 protein fractions (16.5—264 kD) at HH24 at HH26: serum proteins more complex than CSF proteins
				densitometric analysis of the fractions	5 bands -high concentration (constant with GA)
Martín et al. (2006)	Chick	HH25	NA	Western Blot SDS-PAGE	FGF2 isoforms
Parada et al. (2006)	Chick	HH24	NA	2-DE + MS (MALDI-TOF/TOF,ESI–MS/MS)	26 proteins in 8 groups, depending on function
Parada et al., (2008a, 2008b)	Chick	HH20-29	Different GA	HPLC–MS	All-trans retinol—increase from HH20 to HH29
				Slot-blot	RBP (retinol binding protein)—decrease from HH20 to HH29
Mashayekhi et al. (2009)	Chick	E10-21	Different GA	Bradford method	Protein concentration—decreased from E10 to E16, rapid increase on E17—18 then the levels decreased to E21
				Western Blot	NGF (nerve growth factor) presence
				2 site sensitive ELISA	NGF level—decreased from E10 to E16, rapid increase on E17—18 then the levels decreased to E21
Vera et al. (2013)	Chick	HH23-34	Different GA	Western Blot	SCO-spondin is present from HH23 (four bands), in later stages additional 3 bands appear
Vera et al. (2015)	Chick	HH23-30	NA	Sudan Black stain and electrophoresis	LDL and HDL present, LDL > HDL
				Sudan Black stain and electrophoresis + Western Blot (anti SCO-spondin Ab on migrated fragments)	LDL and SCO-spondin—the same migration pattern
				Coimmunoprecipitation assays	LDL and SCO-spondin form a complex in E-CSF
Bachy et al. (2008)	Mouse and Chick	Mouse: E12.5 chick: HH20	Mouse E-CSF vs chick E-CSF	Electronic microscopy	3 types of particles (20 nm—lipoproteins, 35, 50 nm)—different proportion chicks vs mouse
				Western Blot	-chick: ApoA1 (lipoprotein marker), Tsg101 (exosome marker), no FGF8, SHH, BMP4 found -mouse: ApoE (lipoprotein marker)
Parada et al. (2008a)	Chick	HH20-27	Chick adult plasma vs E-plasma vs E-CSF (different GA)	Ultracentrifugation micromethod based on density differences + Sudan Black stain and electrophoresis	-cholesterol and TG: E-CSF < plasma -HDL and proteins: E-CSF, E-plasma < adult plasma -all lipid fractions in E-CSF: increase from HH20 to HH27
	Mouse	E12.5	WT and LDLR KO mice adult plasma vs E-plasma vs E-CSF	Sudan Black stain and electrophoresis	- for both KO and WT: all lipid fractions: E-CSF and E-plasma < adult plasma LDL = the major LP for E-CSF and E-plasma - KO > WT: cholesterol, phospholipids (E-CSF, E-plasma)
Marzesco et al. (2005)	Mouse	E7-E12.5	Different GA	Immunofluorescence microscopy	Prominin 1 containing membrane particles (600 nm and 50–80 nm): increase from E10.5 to E12.5, than rapid decrease to E13.5 Different from exosomes (size and markers)
Huang et al. (2010)	Mouse	E12.5–15.5	Different GA	ELISA	SHH protein (100–300 pg/mL)—no significant differences by GA
Johansson et al. (2013)	Mouse	E12-E14	Gdf7Cre/Otx2^fl/fl^ mice vs controls WT mice (different GA)	Bradford method	Protein concentration: Gdf7-Cre/Otx2^fl/fl^ > WT for E13,14 but not E12
				Western Blot	WNT4 and TGM2 concentration: Gdf7-Cre/Otx2^fl/fl^ > controls for E13
Chau et al. (2015)	Mouse	E10.5–14.5	AF—E8.5, 10.5, 14.5 vs E-CSF- E10.5, E14.5	BCA kit—total protein concentration	E8.5 AF > E10.5 AF > E14.5 AF E8.5 AF > E10.5 CSF, and E14.5 CSF > > E10.5 CSF
				Silver stain—protein complexity	highest at E8.5 AF, regionalized proteome (lateral vs 4th ventricle) at E10.5
				Quantitative LC–MS/MS GProX, David Web Tool—for protein classification	225 proteins = common to all 3 -E8.5 AF = 764 proteins -E10.5 CSF = 504 proteins -E14.5 CSF = 410 proteins 6 clusters of proteins: different during development
				ELISA—for SHH, BOC, GAS1 Sensitive cell lines—BMP, RA activity Immunoblotting- LIFR identification	- SHH (peaked at 14.5), BOC and GAS1, - Highest activity: BMP (at E8.5), retinoic acid (at E14.5) - LIFR in E10.5 after removal of N-glycase (+ PNG-ase)
** (**Tsukada et al. (2015)	Mouse	E12.5–13.5	1.E12.5 vs E13.5 normal CSF	Sandwich ELISA	1. LIF levels: E12.5 CSF < E13.5 CSF no IL6 detected
			2.E12.5, E13.5 CSF normal mice vs dams injected (at E12.5) with rLIF		2. LIF levels E12.5 CSF: control similar to rLIF injected dams E13.5 CSF: control < rLIF injected dams
			+ model of maternal inflammation		

Ab = antibody, AF = amniotic fluid, AFP = alpha fetoprotein, BMP = bone morphogenetic protein, BOC = brother of Cysteine dioxygenase, CS = Carnegie Stage, CSF = cerebrospinal fluid, E = gestational day, E-CSF = embryonic cerebrospinal fluid, E-plasma = embryonic plasma, ELISA = enzyme-linked immunosorbent assay, EGF = epidermal growth factor, ESI–MS/MS = electrospray ionization mass spectrometry/mass spectrometry, FGF = fibroblast growth factor, GA = gestational age, GAS = growth arrest-specific, HDL = high-density lipoproteins, HH = Hamburger-Hamilton stage, HPLC = high performance liquid chromatography, HTx = hydrocephalic, IGF = insulin-like growth factor, KO = knock-out, LC = liquid chromatography, LIF = leukemia inhibitor factor, LIFR = LIF receptor, LDL = low-density lipoproteins, LDLR = low-density lipoprotein receptor, MALDI-TOF/TOF = matrix assisted laser desorption/ionization time of flight/time of flight, miRNA = micro RNA, MS = mass spectrometry, NGF = nerve growth factor, NMRI = Naval Medical Research Institute, NSCs = neural stem cells, RNA = ribonucleic acid, qPCR = quantitative PCR, RA = retinoic acid, rLIF = recombinant leukemia inhibitory factor, RT-PCR = reverse transcription polymerase chain reaction, SHH = sonic hedgehog, SDS-PAGE = sodium dodecyl-sulfate polyacrylamide gel electrophoresis, TG = triglycerides, TGM = transglutaminase, VLDL = very-low-density lipoproteins, WT = wild-type, 2-DE = 2 dimensional gel electrophoresis, RBP = retinol binding protein, SHH = sonic hedgehog, SCO = subcomissural organ, WNT = wingless/integrated protein

**Table 2:** Description of studies regarding the effects of chick embryonic cerebrospinal fluid on cell cultures, tissues or in vivo

CHICK E-CSF (HH20-34)							
A. In ovo protocols							
Author/year	Title	Groups and comparator	Method	Results—cell proliferation	Results—apoptosis	Results—neural differentiation	Other results
Mashayekhi and Salehi (2006)	The importance of cerebro-spinal fluid on neural cell proliferation in developing chick cerebral cortex	CSF-drained chick brains same age controls E10 = drainage, E13 = analysis	Cell proliferation: BrdU + Morphology: methyl green pyronine, hematoxylin–eosin staining	In GE, IZ and CP CSF-drained < controls	NA	NA	Thickness of cortex and of GE: CSF-drained < controls
Salehi and Mashayekhi (2006)	The role of cerebrospinal fluid on neural cell survival in the developing chick cerebral cortex: an in vivo study	CSF-drained chick brains same age controls, E10 = drainage, E13 = analysis	Cell death: TUNEL assay Morphology: methyl green-pyronine staining	NA	GE, IZ, CP- CSF drained > controls	NA	Thickness of cortex: CSF-drained < controls
Bachy et al. (2008)	The particles of the embryonic cerebrospinal fluid: How could they influence brain development?	Embryos injected with antagonists vs controls E3 = injection, E4-5 = analysis	Anti FGF8, anti SHH, anti BMP4 released in chick E-CSF—> cortical development evaluation	NA	NA	NA	No cortical development modification induced by antagonists
M. Alonso et al. (2014)	Retinoic Acid, under Cerebrospinal Fluid Control, Induces Neurogenesis during Early Brain Development	Control In ovo HH21 brain + antiRBP mesencephalic cavity microinjection	Cell proliferation: BrdU + Neurogenesis: Beta3 tubulin Apoptosis: TUNEL assay RA activity: F9-1.8 + cells/area after exposure to X-gal solution	No difference	AntiRBP > control	AntiRBP < control	Reduced or absent RA activity in CSF + antiRBP
Martín et al. (2006)	FGF2 plays a key role in embryonic cerebrospinal fluid trophic properties over chick embryo neuroepithelial stem cells	HH20 = injection in mesencephalic cavity, HH23 = analysis 1. PBS (- control) 2. AFGF2 3. FGF2 4. ANGF	Cell proliferation: BrdU + Neurogenesis: Beta3 tubulin	ANGF, FGF2 similar to PBS AFGF2 < PBS	NA	ANGF similar to control AFGF2, FGF2 < control	AFGF2 only in E-CSF, not in brain tissue after injection in mesencephalic cavity

B. In vivo: adult rat sciatic nerve repair				
Author/year	Title	Groups and comparator	Method	Results
Ghavami et al. (2017)	Functional Recovery of Transected Peripheral Nerve by Means of Microwave Irradiated Collagen Nerve Guides Filled With Chick Embryonic Cerebrospinal Fluid in Rats	- reversed autograft, - collagen nerve conduit filled with E-CSF, - collagen nerve conduit filled with normal saline (NS) - sham surgery	- sciatic functional index (SFI), (day 7,21, 35, 49, 60, 90 after surgery) - electrophysiology, (day 30, 90 after surgery) - histopathology (day 28, 56 after surgery)	SFI day 49 and 60: collagen + E-CSF, autograft > collagen NS Electrophysiology—nerve conduction velocity day 90: collagen + E-CSF, autograft > collagen + NS Histology—regenerated nerve maturation day 90: collagen + E-CSF, autograft > collagen + NS No significant difference between: collagen + E-CSF and autograft

C. In vitro protocols—embryonic chick mesencephalic explants with/without Isthmic Organizer (IsO) HH19-24							
Author/year	Title	Groups and comparator	Method	Results—cell proliferation	Results—apoptosis	Results—neural differentiation	Other results
*C.1. FGF2—comparator*							
Martín et al. (2006)	FGF2 plays a key role in embryonic cerebrospinal fluid trophic properties over chick embryo neuroepithelial stem cells	DM (- control) DM + 1/7 E-CSF (+ control) DM + E-CSF + AFGF2 ± FGF2 DM + FGF2 DM + E-CSF + ANGF	Apoptosis: TUNEL assay, Neurogenesis: Beta3 tubulin	NA	DM + FGF2 = DM > DM + E-CSF	DM + E-CSF + AFGF2 = DM DM + E-CSF + AFGF2 + FGF2 and DM + E-CSF + ANGF similar to DM + E-CSF DM < DM + FGF2 < DM + E-CSF	NA
Parada et al., (2005a, 2005b)	Embryonic cerebrospinal fluid collaborates with the isthmic organizer to regulate mesencephalic gene expression	Basal medium (BM) BM + E-CSF BM + FGF2	Cell proliferation: BrdU + Neurogenesis: Beta3 tubulin, Gene expression: *Fgf8*, *Otx2*, *Shh*, *En1*	Explants without IsO: BM < BM + E-CSF BM < BM + FGF2	NA	Explants without IsO: BM < BM + E-CSF, BM < BM + FGF2	BM: no *Fgf8*, *Otx2*, *Shh* BM + E-CSF: *Otx2* and -with IsO: *Shh*, *Fgf8* -without IsO: ectopic *Shh* BM + FGF2: *Otx2*
Alonso et al. (2011)	Cerebrospinal Fluid Control of Neurogenesis Induced by Retinoic Acid During Early Brain Development	DM = -control, + E-CSF = + control DM + FGF2 DM + all-trans retinol + RBP DM + RA ± FGF2 DM + E-CSF + antiRBP Ab	Neural differentiation: Beta3 tubulin	NA	NA	a.DM = E-CSF + antiRBP Ab b. E-CSF = RA + FGF2 c. DM + FGF2 = DM + RA = DM + all-trans retinol + RBP - a < c < b	NA
		F9-1.8 cells + mesencephalic cells					
		DM = —control, DM + E-CSF = + control F9-1.8 cells alone: DM + E-CSF/RA (different concentrations) F9-1.8 + IsO cells: DM + all-trans retinol ± RBP DM + E-CSF + antiRBP Ab F9-1.8 + mesencephalic cells: DM + all-trans retinol + RBP	RA activity: number of F9-1.8 positive cells (mouse terato-carcinoma)/area after being exposed to X-gal solution	Results RA activity: -F9-1.8 cells: increases with the concentrations of both E-CSF and RA -F9-1.8 cells + IsO cells: DM similar to DM + all-trans retinol DM < DM + E-CSF + antiRBPAb < DM + E-CSF < DM + all-trans retinol + RBP - F9-1.8 cells + mesencephalic cells (without IsO): DM similar to DM + all-trans retinol + RBP DM < DM + E-CSF DM similar to DM for F9-1.8 + IsO cells			
*C.2. Other designs*							
Gato et al. (2005)	Embryonic cerebrospinal fluid regulates neuroepithelial survival, proliferation, and neurogenesis in chick embryos	FCS medium DM (serum free-medium) E-CSF in ovo	Cell proliferation: BrdU + cells, Apoptosis: TUNEL assay, Neurogenesis: Beta3 tubulin	FCS similar to in ovo E-CSF, DM < in ovo	E-CSF < DM,	E-CSF similar to in ovo FCS, DM < in ovo	NA
Parada et al. (2008b)	Low-density Lipoproteins From Embryonic Cerebrospinal Fluid Are Required for Neural Differentiation	DM = —control, DM + E-CSF, in ovo = + control DM + LDF (lipoprotein-depleted fraction) DM + VLDL/LDL/HDL DM + VLDL/LDL/HDL + LDF	cell proliferation: Phosphohistone H3 apoptosis: active caspase3 Neurogenesis: Beta3 tubulin	VLDL/LDL/HDL > DM VLDL/LDL/HDL + LDF or LDF similar to DM E-CSF > all conditions	VLDL/LDL/HDL + LDF or LDF similar to E-CSF < DM LDL/VLDL/HDL similar to DM	VLDL/LDL/HDL similar to E-CSF (similar to in ovo) LDF, HDL/VLDL + LDF similar to DM DM < LDL + LDF < E-CSF	NA
Castells et al. (2012)	Homeostasis of cerebrospinal fluid has a role in early brain development	DM (- control), E-CSF (+ control) Ovalbumin (OVA) BSA (Bovine serum albumin) OVA + αOVA (anti-OVA Ab) Amino-acid mix	Cell proliferation—PhosphohistoneH3 Apoptosis—Active caspase3, Neurogenesis—B3 tubulin	All conditions > DM All conditions < E-CSF All conditions = similar rates	All conditions < DM, All conditions > E-CSF	All conditions > DM, All conditions < E-CSF	NA
Vera et al. (2013)	SCO-spondin from embryonic cerebrospinal fluid is required for neurogenesis during early brain development	**Gain of function:** DM vs DM + SCO spondin **Loss of function:** E-CSF vs E-CSF + anti-SCO spondin Ab	Cell proliferation—BrdU + Apoptosis—active caspase 3, Neurogenesis—Beta3 tubulin	DM + SCO spondin < DM E-CSF < (3x) E-CSF + antiSCO Ab	DM + SCOspondin < (3x) DM, E-CSF < (3x) E-CSF + antiSCO Ab	DM + SCO spondin > (5x) DM E-CSF > (4x) E-CSF + antiSCOAb	NA

Ab = antibody, AF = amniotic fluid, AFGF = anti-fibroblast growth factor antibody, ANGF = anti-nerve growth factor antibody, BMP = bone morphogenic protein, BrdU = bromodeoxyuridine, CP = cortical plate, CSF = cerebrospinal fluid, E = gestational day, E-CSF = embryonic cerebrospinal fluid, DM = Dulbecco’s Modified Eagle Medium, FCS = fetal calf serum, FGF = fibroblast growth factor, GA = gestational age, GE = germinal epithelium, HDL = high-density lipoproteins, HH = Hamburger-Hamilton stage, IZ = intermediate zone, LDL = low-density lipoproteins, NA = not assessed, NSCs = neural stem cells, PBS = phosphate-buffered saline, RA = retinoic acid, RBP = retinol binding protein, SCO = subcomissural organ, SHH = sonic hedgehog, TUNEL = terminal deoxynucleotidyl transferase dUTP nick-end labelling, VLDL = very-low-density lipoproteins.

**Table 3:** Description of studies regarding the effects of mouse embryonic cerebrospinal fluid on cell cultures or tissues

MOUSE E-CSF								
Author/year	Title	GA	Culture type	Groups and comparator	Method for effect evaluation	Results—cell proliferation	Results—neural differentiation	Other results
*A. Embryonic mouse brain tissue and cells*								
Johansson et al. (2013)	The transcription factor Otx2 regulates choroid plexus development and function	E12-E14	Wild-type mouse cortical explants E13	DM 10% E-CSF of Gdf7-Cre/Otx2^fl/fl^ 10–15%E-CSF of WT	MTS assay	10% E-CSF of Gdf7-Cre/Otx2^fl/fl^ > 10–15% E-CSF of WT	NA	NA
Feliciano et al. (2014)	Embryonic Cerebrospinal Fluid Nanovesicles Carry Evolutionarily Conserved Molecules and Promote Neural Stem Cell Amplification	E14	12.5 days mouse NSCs and neuroblasts culture	E-CSF DM E-CSF + rapamycin nanovesicle depleted E-CSF nanovesicles	Phosphorylation of the ribosomal protein S6 (serine S240/244) = readout of IGF-mTORC1 pathway	Nanovesicles had no effect	Nanovesicles had an effect (data not shown)	Phosphorylation - E-CSF > DM (66%), —the increase—ablated by rapamycin (mTORC1 inhibitor) —nanovesicles depleted E-CSF > DM (15%),
Chau et al. (2015)	Progressive Differentiation and Instructive Capacities of Amniotic Fluid and Cerebrospinal Fluid Proteomes following Neural Tube Closure	E10.5	SOX2-EGFP mouse explants: -E8.5, E10.5 forebrain ectoderm - E10.5 olfactory placode	Effect on explants: -E8.5 AF vs -E10.5 CSF vs -E10.5 AF pair-cell assay: -E10.5 CSF -LIF -E10.5 CSF + LIF inhibitors (receptor blocker and neutralizing Ab)	Microscopy evaluation of fluorescent cells (SOX2-fold expression, TUJ expression) -Progenitors SOX2 + and TUJ- -Transient SOX2 + and TUJ + -Neuron SOX2- and TUJ +	SOX2 expression: -*E8.5 explants*: E8.5-AF > E10.5-CSF - *E10.5 explants*: E8.5-AF < E10.5-CSF - *E10.5 olfactory explant:* E8.5-AF < E10.5-AF	Pair-cell assay: E10.5 CSF (20%) similar to LIF progenitors > transient neurons E10.5 CSF + LIF inhibitors (fewer progenitors) < E10.5CSF	NA
*B. Adult mouse brain tissue and cells*								
Carnicero et al. (2013)	Embryonic Cerebrospinal Fluid Activates Neurogenesis of Neural Precursors within the Subventricular Zone of the Adult Mouse Brain	E12.5	Adult mouse neural precursors from SVZ	DM DM + E-CSF DM + adult CSF	Cell proliferation: BrdU + cells, Neurogenesis: Beta3 tubulin, 1. BrdU + Beta 3 tubulin low level = newborn neurons, 2. BrdU + Beta 3 tubulin high level = young neurons 3. only BrdU = neural precursor	NA	Neural precursors: DM similar to E-CSF Newborn neurons: adult CSF < DM = E-CSF Young neurons: adult CSF < DM < E-CSF (38%)	NA
Alonso et al. (2017)	Embryonic Cerebrospinal Fluid Increases Neurogenic Activity in the Brain Ventricular-Subventricular Zone of Adult Mice	E12.5	Adult mouse organotypic culture of SVZ,	Latex microbeads soaked in: - E-CSF - DM - adult CSF	Nissl bodies Cell proliferation, migration: BrdU + cells BrdU + SOX2—undifferentiated NSC BrdU + Neurod2—early-stage neuron BrdU + Beta3 tubulin—young neurons (low/high levels) BrdU + Calretinin—differentiated neurons BrdU + Doublecourtin—migratory NSC	BrdU: E-CSF > DM, adult CSF BrdU + SOX2: E-CSF > DM (141%)	BrdU + Neurod2: E-CSF > DM (78%) BrdU + Beta3 tubulin: - E-CSF > DM (34% for total), 69% were high level, - adult-CSF < DM (10% for total), 67,5% were low level BrdU + Calretinin: E-CSF > DM (61%)	Migratory pattern: (in proximal segment of RMS): E-CSF > DM (127%)
Madrigal et al. (2023)	Embryonic cerebrospinal fluid influence in the subependymal neurogenic niche in adult mouse hippocampus	E13.5	Adult mouse organotypic cultures hippocampal zone (SEZ, DMS and hilus)	For SEZ, DMS, hilus—latex microbeads soaked in: - E-CSF - DM		BrdU + SOX2: for all 3 areas E-CSF > controls	BrdU + Beta3 tubulin: -for SEZ, DMS: E-CSF > controls -controls: SEZ < DMS < hilus -E-CSF: SEZ = DMS = hilus BrdU + Calretinin: **-**for all 3 areas E-CSF > controls -controls: SEZ = DMS = hilus -E-CSF: SEZ < DMS < hilus	Migratory pattern BrdU + Doublecortin: -for all 3 areas E-CSF > controls - E-CSF and control: DMS < SEZ < hilus

Ab = antibody, AF = amniotic fluid, BrdU = bromodeoxyuridine, CSF = cerebrospinal fluid, DM = Dulbecco’s Modified Eagle Medium, DMS = Dentate Migratory Stream, E = gestational day, E-CSF = embryonic cerebrospinal fluid, FBS = fetal bovine serum, GA = gestational age, Gdf = growth differentiation factor, Gdf7-Cre/Otx2^fl/fl^ = Gdf7-Cre mouse line with an Otx2 deletion, IGF = insulin-like growth factor, NA = not assessed, NSCs = neural stem cells, RMS = Rostral Migratory Stream, SVZ = subventricular zone, SEZ = Sub-ependymal Zone, TUNEL = terminal deoxynucleotidyl transferase dUTP nick-end labelling, WT = wild-type

**Table 4:** Description of studies regarding the effects of rat embryonic cerebrospinal fluid on cell cultures or tissues

RATS E-CSF								
A. Neural tissues and cells								
Author/year	Title	GA	Culture type	Groups and comparator	Method for effect evaluation	Results—cell proliferation	Results—apoptosis	Results—neural differentiation
*A.1. Embryonic tissue*								
Martin et al. (2009)	Early embryonic brain development in rats requires the trophic influence of cerebrospinal fluid	E13.5	E13.5 rat mesencephalic explants	Controls in vivo: initial- 12.5, final-13.5 days vs 24 h culture in: -DM -DM + 15% E-CSF -DM + FCS	Cell proliferation: BrdU + cells Apoptosis: TUNEL assay Neural differentiation: Beta3 tubulin + cells	DM < FCS < E-CSF < final control initial < final control	DM > FCS > E-CSF > controls	DM and FCS < E-CSF < final control initial control < final control
Lamus et al. (2020)	FGF2/EGF contributes to brain neuroepithelial precursor proliferation and neurogenesis in rat embryos: the involvement of embryonic cerebrospinal fluid	E13.5	E13.5 rat mesencephalic explants	A. control in vivo vs B. explants cultured in 1. DM 2. DM + 15% E-CSF 3. DM + E-CSF + AFGF2 4. DM + E-CSF + AEGF 5. DM + FGF2 ± AFGF2 6. DM + EGF ± AEGF	Cell proliferation: BrdU + cells Neurogenesis: Beta3 tubulin + cells	a.DM, EGF < E-CSF < FGF2 b. FGF2 + AFGF2 < E-CSF + AFGF2 < E-CSF	NA	DM similar to in vivo control DM < E-CSF similar to FGF2 FGF2 > E-CSF (close to the luminal surface) E-CSF > E-CSF/FGF2 + AFGF2, E-CSF > E-CSF + AEGF
Miyan et al. (2020)	Subarachnoid cerebrospinal fluid is essential for normal development of the cerebral cortex	E19.5	E19.5 rat brain slices	1. latex beads soaked in PBS (control) vs in E-CSF placed on pial vs ventricular surface vs on both surfaces 2. brain slices cultured in PBS vs in E-CSF	Cell proliferation: BrdU + cells, Optic microscopy (+ migration) Apoptosis: TUNEL assay	1.—PBS beads—NO -E-CSF beads on pial surface—NO —E-CSF beads on ventricular or on both surfaces—YES 2. E-CSF > PBS medium	E-CSF < PBS	NA
Yari et al. (2013)	Effect of embryonic cerebrospinal fluid on proliferation and differentiation of neuroprogenitor cells	E16, E18, E20	E15.5 rat SVZ neurospheres	Control: No exposure, Exposure to E16 CSF Exposure to E18 CSF Exposure to E20 CSF	Cell proliferation: MTT assay Neurogenesis: anti-GFAP (for astrocytes) Neurosphere size	Control similar to E20 < E16 and E18	NA	Anti-GFAP: control similar to E20 > E16 and E18, Neurosphere size: control similar to E20 < E16 and E18
*A.2. Adult tissue*								
Nabiuni et al. (2012)	In vitro effects of fetal rat cerebrospinal fluid on viability and neuronal differentiation of PC12 cells	E17, E18, E19, E20	Rat PC12 cell cultures— pheochromo-cytoma	DM vs DM + E-CSF (E17, E18, E19, E20) vs DM + bFGF (positive control)	Cell proliferation: MTT assay Neural differentiation: neurite length (phase of contrast microscopy) Beta3 tubulin, MAP2	E-CSF > DM E18-CSF the greatest value	NA	Morphological differentiation: - present: bFGF, E17,19 CSF Beta3 tubulin: -present: for bFGF, E17,19,20SF MAP2:- NO: for DM, E18,20 CSF - in neurites: bFGF, E19 CSF - in neurites, cell bodies:E17 CSF
Peirouvi et al. (2015)	High neuronal/astroglial differentiation plasticity of adult rat hippocampal neural stem/progenitor cells in response to the effects of embryonic and adult cerebrospinal fluids	E13.5 E17	Rat NSCs (hipp-NS/PCs from adult rats)	DM ± 7%FCS (control) vs DM + E13.5/17 CSF (15, 20%) vs DM + adult CSF (15, 20%) *for all normal KCl vs high KCl	Cell proliferation: MTT assay, sRT-PCR: Ki67, nestin, SOX2 Neurogenesis: immunochemistry: antiGFAP -astrocytes, antiMAP2-neurons sRT-PCR for RNA: -Beta 3 tubulin = neuronal -GFAP = astroglial	MTT assay: Adult CSF < control < ECSF E-CSF 20% > 15% (for E13.5) Ki67, nestin, SOX2: high KCl > normal KCl	NA	Immunochemistry: - in E-CSF: MAP2 > GFAP -MAP2 (in E-CSF): high > normal KCl - adult CSF: MAP2 < GFAP sRT-PCR: -Beta 3 tubulin: adult CSF < control < E-CSF -GFAP: adult CSF > control

Author/year	Title	GA	Culture type	Groups + comparator	Method for effect evaluation	Results-cell proliferation	Results—neural differentiation
*B. Mesenchymal stem cells*							
Shokohi et al. (2018b)	Fetal Cerebrospinal Fluid Promotes Proliferation and Neural Differentiation of Stromal Mesenchymal Stem Cells Derived from Bone Marrow	E17, 18,19	Adult mouse BM-MSC	Negative control vs E17 CSF (3,7,10%) vs E18 CSF (3,7,10%) vs E19 CSF (3,7,10%) vs bFGF (positive control)	Cell proliferation: MTT assay Neurogenesis: - morphology, - neurite growth - Immunochemistry (anti -MAP2/Beta3 tubulin)	E17,18 CSF > E19 CSF E-CSF > control, E-CSF:10% > 3% or 7%	Morphology: E-CSF, bFGF > control Neurite growth: bFGF > E17, E19 CSF > E18 CSF, control Immunochemistry: bFGF = E17,19 > E18 > control
Shokohi et al. (2018a)	In Vitro Effects of Wistar Rat Prenatal and Postnatal Cerebrospinal Fluid on Neural Differentiation and Proliferation of Mesenchymal Stromal Cells Derived from Bone Marrow	E19, E20	Adult mouse BM-MSC	Negative control vs E19 CSF (3,7,10%) vs E20 CSF (3,7,10%) vs P1 (postnatal) CSF vs bFGF (positive control)	Cell proliferation: MTT assay Neurogenesis: - morphology, - neurite growth - Immunochemistry (anti-MAP2/Beta3 tubulin)	E-CSF > control, E-CSF:10% > 3% or 7%	Morphology: E19 CSF > E20, P1 CSF > control Neurite growth: bFGF > E19 CSF > E20,P1 CSF > control Immunochemistry: bFGF > E-CSF, highest E-CSF = E19 CSF
Dorazehi et al. (2018)	Potential Use of Amniotic Membrane—Derived Scaffold for Cerebrospinal Fluid Applications	E17	Adult rat BM-MSC, on human decellularized amniotic membrane	E-CSF 7% vs bFGF vs non-treated cells	Cell proliferation: MTT assay Neurogenesis: electron microscopy, immunochemistry (anti-MAP2/ Beta3tubulin)	increase from day 5 to 15, followed by a decrease until day 30 (but day 30 > day 1)	Electronic microscopy: E-CSF similar to non-treated Immunochemistry: - E-CSF > bFGF -no markers: in non-treated cells
Mohammadi-Mahdiabadi-Hasani et al. (2020)	The Effects of Embryonic Cerebrospinal Fluid on The Viability and Neuronal Differentiation of Adipose Tissue-Derived Stem Cells in Wistar Rats	E17, E18,E19	Adult rat adipose derived stem cells (ADSC)	DM vs DM + 10% E-CSF (E17, 18, 19) vs Beta ME (+ control)	Cell Proliferation: MTT assay Neurogenesis: microscopy—neurite outgrowth, immunochemistry (anti-beta3tubulin)	Beta ME < DM < E-CSF	Neurites: present in E18, E19—CSF, absent in E17 CSF and DM Beta 3 tubulin: all ages of E-CSF > DM,
Goudarzi et al. (2020)	Role of cerebrospinal fluid in differentiation of human dental pulp stem cells into neuron-like cells	E19	Human dental pulp stem cells (hDPSC)	DM vs DM + 5% E-CSF	Cell proliferation: MTT assay, RT-PCR: stemness (oct4, Sox2), Neurogenesis: microscopy (morphology, neurites length, crystal violet-Nissl bodies, silver stain-neurites) immunochemistry—Nestin—progenitor, MAP2—mature RT-PCR Nestin -progenitor, NF-M differentiating, NF-H mature motor neurons, internal control (GAPDH)	RT-PCR: (oct4, sox2): 1. high at first, progressively decreased, 2. the decrease in E-CSF > DM	Microscopy: -morphology (day 8): E-CSF treated = neuron morphology + Nissl bodies, control = fibroblast morphology -Neurite length (day4, 8): E-CSF > > control Immunochemistry: -Nestin: E-CSF > control -*MAP2: only in E-CSF* RT-PCR: -nestin: 1. in E-CSF group: high at first, progressively decrease 2. at first E-CSF > control, then E-CSF < control -NF-M: E-CSF > control -*NF-H: only in E-CSF* from day 4, increase progressively

Ab = antibody, AF = amniotic fluid, AEGF = anti-epidermal growth factor antibody, AFGF = anti-fibroblast growth factor antibody, Beta ME = beta mercaptoethanol, (b)FGF = (basic) fibroblast growth factor, BM-MSCs = bone marrow mesenchymal stem cells, BrdU = bromodeoxyuridine, CSF = cerebrospinal fluid, DM = Dulbecco’s Modified Eagle Medium, E = gestational day, E-CSF = embryonic cerebrospinal fluid, EGF = epidermal growth factor, FCS = fetal calf serum, GA = gestational age, GFAP = glial fibrillary acidic protein, hipp-NS/PCs = hippocampal neural stem/progenitor cells, NA = not assessed, NSCs = neural stem cells, PBS = phosphate buffered saline, RNA = ribonucleic acid, (s)RT-PCR = (single-step)reverse transcription polymerase chain reaction, SVZ = subventricular zone, TUNEL = terminal deoxynucleotidyl transferase dUTP nick-end labelling,

## Results and Discussion—Studies Description

### The Composition of Embryonic CSF

From our search, we only identified three studies using human E-CSF, all investigating its composition (Christiansen et al., 2000; Feliciano et al., 2014; Zappaterra et al., 2007). Taking into account the importance of the subject, we decided to present them separately. The small number of published studies using human E-CSF is likely due to reduced sample availability, ethical concerns, and the unpredictability of pregnancy termination. In order to overcome these barriers and respect the ethical rights of the participants, international organizations, such as American Medical Association (AMA), have issued recommendations for fetal tissue research (Joint Report of the Council on Ethical and Judicial Affairs & and the Council on Scientific Affairs, n.d.).

The first study, conducted by Christiansen et al. (2000), used immune-electrophoresis to analyze the presence and relative concentrations of alpha-fetoprotein and albumin in the CSF and serum of six human fetuses (17, 20, 22, and 23 weeks gestational age). Although the levels of the investigated proteins were constant, the CSF/serum ratio decreased with gestational age. Even though the study does not offer a complex view of the issue, it is one of the first studies to raise the question about the proteins present in E-CSF, their roles, and the differences that arise at different gestational ages.

Using mass spectrometry, a much more specialized method, Zappaterra et al. (2007) conducted the first proteomic description of the human E-CSF (using one human embryo, Carnegie Stage 20) in comparison to the rat E-CSF. The group was able to identify 188 proteins in human E-CSF (the majority being secreted proteins), of which 83 were common to rat E-CSF. Even though they identified a limited number of proteins, compared to the ones identified to date in the adult CSF (3000 proteins (Macron et al., 2018)), it remains the only study up to the present that attempted a proteomic description of human CSF. The limitations of the study were linked to both the method of MS that was available in 2007 and the limited access to fetal tissue, the group having access to only two samples of E-CSF, of which one was contaminated.

In the context of the increasing applications of adult CSF exosomes (Yoo et al., 2018), Feliciano et al. (2014) described the nanovesicles from human and rat E-CSF, using electronic microscopy and genomic techniques. They identified exosome markers on the nanovesicles and insulin-like growth factor (IGF) pathway elements in the particles from both species, at different concentration levels. The group also investigated the presence of microRNAs, the majority common between rat (147) and human (167), with 15 microRNAs having significantly different expressions across species.

Another more recent study, published by Ryan et al. (2024), focused on microRNA from non-human primate E-CSF, finding 77 microRNAs in normal controls and significant differences in tetrahydrocannabinol-exposed fetuses. Further studies are needed to analyze the interspecies differences and also to perform a comparison to adult CSF microRNAs in order to find the significant microRNAs for human neurodevelopment.

Regarding vesicles, Feliciano et al. (2014) were not the only ones to perform an analysis on the E-CSF. We included two other studies that investigated the presence of vesicles in animal E-CSF by microscopy. The first study, published by Marzesco et al. (2005) used immunofluorescence methods in order to identify prominin 1 as a vesicle marker in mice E-CSF. The study compared the load of prominin 1 at different GA, with the highest amount identified at 12.5 days. As opposed to Feliciano et al., the authors stated that the identified particles do not have exosome characteristics. Bachy et al. (2008) conducted the second included study on mice and chicks, comparatively. Using electronic microscopy, they identified three types of particles (different sizes), with a variable interspecies proportion. They also used immunological methods to identify specific markers: ApoA1 for chicks and ApoE for mice, specific for lipoproteins, and Tsg101 in chicks, specific for exosomes. The presence of apolipoproteins is concordant with the findings of Parada et al., (2005a, 2005b) regarding the presence and the variation of these substances in mammalian and avian E-CSF. They tried to identify additional proteins, such as SHH and BMP, through Western Blot, but the method did not detect them, while other studies managed to identify SHH through ELISA (Chau et al., 2015; Huang et al., 2010), and BMP using sensitive cell lines (Chau et al., 2015), showing that, depending on concentration, the method’s sensitivity is also of interest. Even though exosomes from adult tissues are used in the present in diagnostic and treatment, E-CSF vesicles are still in need of investigation in order to define their specific properties and propose further uses.

The study published by Gato et al. (2004) was the first study that focused on E-CSF proteome description. The authors used electrophoresis for protein identification in chick E-CSF. Due to the limitations of the method, it was able to identify only 21 protein fractions, but it also demonstrated an increase in protein concentration with gestational ages (GA) and a higher complexity of serum proteins compared to E-CSF proteins.

There were additional four studies that aimed to describe the protein composition of E-CSF, that used mass spectrometry. The group of Parada et al. investigated, in two consecutive studies, the composition of chick E-CSF (Parada et al., 2006) and rat E-CSF (Parada et al., 2005a, 2005b) using similar methods, and compared the results. They identified 26 proteins in avian E-CSF and 31 proteins in mammalian E-CSF, dividing them into groups based on function. Six groups were common between species: extracellular matrix, proteins of osmotic pressure and ion carriers, apolipoproteins (more complex in rats than chicks), vitamin and corticosteroid carriers, antioxidant and antimicrobial proteins, and proteins with unknown function. Chick E-CSF also presented intracellular proteins and proteins related to cell quiescence and death, while rat E-CSF presented an additional group of enzymes or enzyme regulators (Parada et al., 2005a, 2005b).

Chau et al. (2015) compared mouse E-CSF with amniotic fluid at different GA in terms of protein concentration and complexity, osmolality, and composition. The highest osmolality and protein complexity was observed in early amniotic fluid (before the formation of E-CSF). In the following period, along with E-CSF formation at 10.5 days GA (E10.5), they observed the regionalization of the proteome (between lateral and fourth ventricle). In terms of composition, the study used liquid chromatography-mass spectrometry/mass spectrometry (LC–MS/MS) for proteome description, identifying 225 common proteins, with 764 proteins in early amniotic fluid, 504 proteins in E10.5 CSF, and 410 in E14.5 CSF, divided into 6 clusters. The authors were also able to identify, through immunochemical methods, signaling molecules, such as SHH, BOC, GAS1, and LIFR (that mass spectrometry was not able to identify), and to measure retinoic acid and BMP activity.

The latest study to investigate the proteomic composition of E-CSF was published by Requena-Jimenez et al. (2021) and focused on the differences between normal and hydrocephalic rat E-CSF composition. We extracted the data regarding normal rat E-CSF proteome, the study being able to identify up to 982 proteins in rat E-CSF, significantly more than those identified by Parada et al., (2005a, 2005b) and Zappaterra et al. (2007). The difference is mainly due to the increasing perfecting of the accuracy and sensitivity of mass spectrometry methods, and partly due to the difference of gestational age. The authors used bioinformatic methods to include the identified proteins in known biological pathways, the most important ones being xenobiotic-related.

**Fig. 4:**
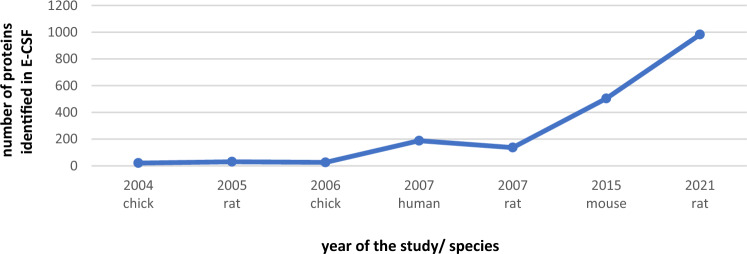
Number of proteins identified in embryonic cerebrospinal fluid in the included studies regarding proteome assessment by year and species

Although the studies of E-CSF proteome show variability of studied species and GA, the number of identified proteins increases with the year of publication. As a majority, they use the same principle (mass spectrometry), the advances in the method made possible the identification of new proteins and a better description of the E-CSF proteome (Fig. 4).

Another important area on which the literature is focused is the relative concentration on lipoproteins and their potential roles. Parada et al. (2008a) described the lipoprotein composition of chick and mouse E-CSF. They investigated the proportions of the different lipoprotein fractions between E-CSF, embryonic plasma, and adult plasma, concluding that low-density lipoproteins (LDL) are the most prominent fraction in E-CSF and that LDL-receptor knock-out mice have an increased level of cholesterol and phospholipids in E-CSF and plasma. Vera et al. (2013) managed to demonstrate the presence of subcomissural organ (SCO)-spondin in chick E-CSF using Western blot. In 2015, the same group conducted a study regarding the interaction between lipoproteins and SCO-spondin in chick E-CSF (Vera et al., 2015). Using co-immunoprecipitation, they showed that LDL and SCO-spondin form a complex in the E-CSF, suggesting their interdependence.

The leukemia inhibitor factor (LIF) is another known developmental factor. Three of the included studies focused on LIF pathways: the LIF-ACTH-LIF pathway (Simamura et al., 2010) and its suppression determined by maternal inflammation (Tsukada et al., 2015), and the LIF-IGF pathway (Takata et al., 2022). We extracted the data coherent with the scope of our review, regarding the composition of E-CSF determined by ELISA. Simamura et al. (2010) demonstrated the presence of LIF in rat E-CSF with a peak at 15.5 days of gestation. The study of Tsukada et al. (2015) showed that, in mice, LIF levels are higher at 13.5 days than 12.5 days gestational age (GA) and external LIF administered to dams was incorporated in E-CSF only at 13.5 days GA. The group was also able to demonstrate that maternal inflammation determined the decrease of LIF levels, but they did not identify interleukin-6 (IL6) in the E-CSF in any condition. In coherence with the data regarding the presence of LIF-IGF pathways, Takata et al. (2022) showed the presence of IGF1 and IGF2 in rat E-CSF, that peaked at E17.5.

The presence of specific substances in the E-CSF was identified through different methods (Table 5), such as ELISA: sonic hedgehog (SHH) protein (Chau et al., 2015; Huang et al., 2010), Western Blot: nerve growth factor (NGF) with the same peak as total protein concentration at E17 (Mashayekhi et al., 2009), wingless/integrated 4 (WNT4) and transglutaminase 2 (TGM2) proteins, in higher concentration in mice with *Otx2* deletion than controls (Johansson et al., 2013), fibroblast growth factor 2 (FGF2) (Lamus et al., 2020; Martín et al., 2006), epidermal growth factor (EGF) (Lamus et al., 2020), retinol binding protein (RBP) (Parada et al., 2008a, 2008b), and mass spectrometry: all- trans retinol (Parada et al., 2008a, 2008b) increases in concentration with GA as RBP decreases. The growth factors identified also represent good candidates for neurogenesis support in vitro or in adult pathological models.

**Table 5:** Summary of identified substances in embryonic cerebrospinal fluid by method of detection, from the included studies

ELISA	Immunoblot	Electrophoresis ± staining	Sensitive cell lines	Mass spectrometry
SHH Chau et al. (2015),Huang et al. (2010) LIF Simamura et al. (2010)**, **Tsukada et al. (2015) IGF1,2 Takata et al. (2022) BOC Chau et al. (2015) GAS1 Chau et al. (2015)	EGF Lamus et al. (2020),FGF2 Lamus et al., (2020), Martín et al. (2006) NGF Mashayekhi et al. (2009), SCO-Spondin Vera et al., (2013, 2015) TGM2, WNT4 Johansson et al. (2013) LIF receptor Chau et al. (2015) RBP Parada et al., (2008a, 2008b)	AFP Christiansen et al. (2000) Albumine Christiansen et al. (2000) HDL, LDL, VLDL Parada et al. (2008a); Vera et al. (2015)	Retinoic acid, BMP Chau et al. (2015)	All trans retinol Parada et al. (2008a, 2008b)

AFP = alpha fetoprotein, BMP = bone morphogenetic protein, BOC = brother of cysteine dioxygenase, ELISA = enzyme-linked immunosorbent assay EGF = epidermal growth factor, FGF = fibroblast growth factor, GAS = growth arrest-specific, HDL = high-density lipoproteins, IGF = insulin-like growth factor, LIF = leukemia inhibitor factor, LDL = low-density lipoproteins, NGF = nerve growth factor, RBP = retinol binding protein, SHH = sonic hedgehog, SCO = subcomissural organ, TGM = transglutaminase, VLDL = very-low-density lipoproteins, WNT = wingless/integrated protein

Five of the included studies also focused on the variation of total protein concentration in the E-CSF with GA using the Bradford method and Bicinchoninic Acid Protein (BCA) assay. The studies used different species. In chick E-CSF, Gato et al. (2004) showed an increase from Hamburger-Hamilton (HH) stage 17 to 30, and Mashayekhi (2009) et al. described a peak at day 17–18 GA. Nabiuni et al. (2012) showed a progressive decrease from day 17 to day 21 in rat E-CSF and Johansson et al. (2013) demonstrated a higher concentration in mice with deleted *Otx2* (transcription factor with role in neurodevelopment) than controls at E13 and E14, while Chau et al. (2015) concluded that, in mice E-CSF, the protein concentration increases with GA. The study of protein concentration, through its simple and available methodology, represents a good starting point for research, as it enables new research teams to choose the best GA for the study design and animal model of interest.

Some of the research groups chose to develop pathological models in the study of E-CSF, such as hydrocephaly (Requena-Jimenez et al., 2021), toxic exposures—tetrahydrocannabinol (Ryan et al., 2024), or maternal inflammation (Tsukada et al., 2015). These studies provide insights regarding the implications of E-CSF in disease development, but the conclusions cannot be widely applied without solid data regarding normal E-CSF composition. It is necessary to study first the normal aspects, in order to correctly understand the abnormal.

### The Effects of Chick Embryonic CSF

#### In Ovo and In Vivo Protocols

Five studies used in ovo protocols, while two additional studies used in ovo development as positive control (Gato et al., 2005; Parada et al., 2008a). There were two types of protocols: E-CSF drainage and E-CSF microinjection. The group of Mashayekhi (Mashayekhi & Salehi, 2006; Salehi & Mashayekhi, 2006) used similar study designs performing E-CSF drainage at 10 days GA, with result analysis after 3 days. They concluded that E-CSF-drained embryos had lower cell proliferation, higher apoptosis, and reduced thickness of the cortex and germinal epithelium. In comparison, Bachy et al. (2008) investigated the effect of injecting antagonists of specific molecules (FGF8, SHH, BMP4) presumed to have a role in neurogenesis in the CSF of 3 days old embryos, but they observed no difference in terms of morphological cortical development between injected brains and controls after 1 day. Using a similar approach, Martín et al. (2006) injected the mesencephalic cavity of HH20 embryos with anti-FGF2 antibody (AFGF2), FGF2 being a presumed factor in neurogenesis. They used phosphate-buffered saline (PBS) injection, and the injection of anti-NGF antibody (ANGF) as control—NGF is presumed not to be implied in this stage of development. ANGF injection did not show differences in terms of cell proliferation or neural differentiation compared to PBS, but the injection of AFGF2 showed a significant reduction in both. AFGF2 was confined only to blocking the FGF2 in the E-CSF, with no traces of the antibody being found in the brain tissue. The study also evaluated the effect of FGF2 injection, that had no beneficial outcome, but a decrease in neural differentiation compared to control. These outcomes suggest that neurogenesis is a fine-regulated process that requires intrinsic FGF2, but it becomes dysregulated by further addition of the factor. M. Alonso et al. (2014) also used a microinjection protocol with anti-RBP antibodies, showing the importance of RBP and retinol pathway in neurogenesis. The antagonist determined an important reduction in retinoic acid secretion in E-CSF, and subsequently higher apoptosis and lower differentiation, with no significant effect on cell proliferation.

The in ovo protocols offer a perspective of the importance of E-CSF in neural development, showing that its presence is necessary (E-CSF drained brains did not develop properly), but from the molecules that are present in E-CSF, only FGF2 has been proved to be important, while FGF8, SHH, BMP4, and NGF showed no implication (antagonist injection did not modify the development).

One study used an in vivo protocol, focusing on adult rat sciatic nerve reconstruction (Ghavami et al., 2017). The researchers showed that repair with collagen nerve conduit filled with E-CSF is non-inferior to reversed autograft, with better results than collagen nerve conduit filled with saline or sham surgery in terms of functional, electrophysiological, and histological recovery. The study supports the idea of regenerative properties of E-CSF even in adult tissues (Ghavami et al., 2017).

The remaining studies used in vitro protocols, culturing embryonic tissues (mesencephalic chick explants) in different media, with a single study using also F9.1–8 cells (Alonso et al., 2011).

#### In Vitro Protocols—FGF2 Role

Three studies focused on the role of FGF2 in vitro, all of them using basal medium as negative control and E-CSF as positive control (Alonso et al., 2011; Martín et al., 2006; Parada et al., 2005a, 2005b). All three studies showed that, in terms of neurogenesis, the addition of FGF2 to the basal medium leads to better results than the basal medium alone, but is inferior to the neurogenetic effect of the E-CSF. Similar results have been obtained regarding apoptosis (Martín et al., 2006) and cell proliferation (Parada et al., 2005a, 2005b). Martín et al. (2006) used FGF2 and AFGF2 to study the implications of the growth factor in neurogenesis and apoptosis. The addition of AFGF2 to E-CSF cancels the neurogenetic effect of the E-CSF, while the addition of ANGF does not, showing the specific implication of the antibody in blocking the process, supporting the results obtained in ovo. Parada et al. (2005b) used explants, with or without the isthmic organizer (IsO), and studied not only the effects on proliferation and apoptosis, but also gene expression after culture. None of the investigated genes were expressed in the cultures grown in basal medium alone, while in those supplemented with FGF2, only *Otx2* was expressed. The explants cultured in E-CSF expressed not only *Otx2*, but also *Shh*. In the presence of the IsO, *Fgf8* and normal *Shh* were expressed, while in the absence of the IsO, the *Shh* was ectopic. The study of Alonso et al. (2011) focused on the effect of both FGF2 and retinoic acid (RA), proving that their addition generated results superior to basal media alone, but inferior to E-CSF regarding neural differentiation. Similar results to RA addition offered the combination of all-trans-retinol and retinol binding protein (RBP). Also, the inhibition of the RBP contained in the E-CSF, by anti-RBP antibody addition, canceled the neurogenetic effect, similar to the addition of anti-FGF2 antibodies studied by Martín et al. (2006). A similar effect was obtained by the same team in the in ovo protocol (2014).

#### Other In Vitro Culture Protocols

Alonso et al. (2011) investigated the importance of retinoic acid using F9-1.8 cells (extracted from mouse teratocarcinoma) as markers of RA activity. They demonstrated that F9-1.8 cells exposed to E-CSF show higher activity (that increases with E-CSF culture concentration). When growing F9-1.8 cells in co-culture with chick mesencephalic cells, the E-CSF had a trophic role, leading to the increase of RA activity. The presence or absence of the IsO did not influence the RA activity of the cells in the basal medium, but the exposure to different components from the retinol pathway lead to different results, depending on the presence of the IsO in co-culture. The exposure to all-trans retinol and RBP lead to high activity in the presence of IsO cells (even higher than exposure to E-CSF), while in the absence of IsO cells, had no effect on RA activity.

The remaining four studies aimed to determine the influence of different conditions on cell proliferation (using *BrdU* positive cells or phosphohistone H3), apoptosis (using TUNEL assay or active caspase 3), and neurogenesis (using Beta 3 tubulin positive cells) in the mesencephalic explants. The study of Gato et al. (2005) showed that while fetal calf serum determined higher cell proliferation, chick E-CSF induced higher neural differentiation, similar to in ovo. Regarding apoptosis, E-CSF cultured explants had fewer apoptotic cells than explants cultured in serum-free medium. In a similar approach, Castells et al. (2012) demonstrated that different additions to the basal medium (such as ovalbumin ± anti-ovalbumin antibodies, bovine serum albumin, and amino-acid mix) improve cell proliferation and neurogenesis and reduce apoptosis, but show results inferior to E-CSF, that seems to have higher qualities as a culture medium for neural progenitors.

According to Parada et al. (2008a), adding different lipoprotein fractions, alone or in combination with lipoprotein depleted fraction (LDF), to the basal medium determines inferior results in terms of cell proliferation compared to E-CSF. The addition of LDL, VLDL, or HDL determined higher proliferation and neurogenesis than basal medium alone or LDF-enriched cultures. The exception is the combination of LDL and LDF, that shows higher differentiation than basal medium, but still is inferior to the E-CSF cultured explants. On the other hand, the addition of LDF determines low apoptosis, similar to E-CSF, that cannot be obtained by culturing the explants in lipoproteins alone. The study shows that LDF inhibits cell proliferation and neurogenesis while promoting cell survival.

Vera et al. (2013) investigated the importance of SCO-spondin using two protocols, a gain of function protocol that showed better cell proliferation, lower apoptosis, and higher neurogenesis when adding SCO-spondin to the basal culture medium, and a loss of function approach that showed that inhibiting the SCO-spondin present in the E-CSF (by means of specific antibodies) leads to lower cell proliferation and neurogenesis and higher apoptosis.

Due to lower costs and higher availability, the avian model has been widely used for E-CSF research. Even though there is a difference of composition between avian and mammal CSF (Parada et al., 2005a, 2005b), these studies provide useful hypotheses that can be further tested on other species, even human.

### The Effects of Mouse Embryonic CSF

#### Embryonic Brain Tissue

Three of the included studies used embryonic mouse brain cells: E13 cortical explants (Johansson et al., 2013), E12.5 neural stem cells (Feliciano et al., 2014) and E8.5-E10.5 forebrain, and olfactory explants (Chau et al., 2015).

The study of Johansson et al. (2013) focused on the importance of the *Otx* transcription factor, showing higher cell proliferation in explants cultured in mutant E-CSF (with deleted *Otx* factor) than wild-type E-CSF (regardless of 10 or 15% concentration). The expression of *Otx* was also observed in chick explants cultured in E-CSF by Parada et al. (2005b). Using different protocols, both studies proved that *Otx* is involved in cellular processes during brain development.

The group of Feliciano et al. (2014) aimed to characterize the role of the nanovesicles present in E-CSF. The authors noted that nanovesicles had determined an increase in neurogenesis and no effect on cell proliferation, but data for these findings were not shown in the cited study. They also investigated the IGF-mTORC1 pathway using the phosphorylation of the ribosomal protein S6, that had a 66% higher concentration when cultured in the presence of E-CSF than basal medium. The difference was annulled by the addition of rapamycin (inhibitor of mTORC1) and decreased to only 15% when the used E-CSF was depleted on nanovesicles.

Chau et al. (2015) investigated the influence of different gestational ages E-CSF and amniotic fluid on neurogenesis, using *Sox2* and *Tuj* expression. They showed that the differences are dependent on both the age of the explants and the fluids (E-CSF and amniotic fluid), and for superior results in vitro in terms of neural differentiation, the age of the explant should be matched with the age of the fluid. By means of pair cell assay, the group demonstrated that the presence of leukemia inhibitor factor (LIF) and the presence of 20% E-CSF in culture medium lead to similar results, respectively higher progenitor proliferation, detrimental to transient cells or neurons. The addition of inhibitors of the LIF pathway to E-CSF leads to a decrease in progenitor cells.

#### Adult Brain Tissue

Carnicero et al. (2013) isolated neural progenitor cells from the subventricular zone of adult mice and cultured them in CSF (adult and embryonic). They evaluated neurogenesis by the presence of cells in different stages of development (neural precursors, newborn neurons, and young neurons). There was no difference between culture conditions regarding the number of neural precursors, but there were significantly fewer newborn and young neurons in adult CSF than in basal medium. E-CSF presence increased the number of young neurons, but not of newborn neurons, compared to basal medium. The study shows the role of E-CSF in the neurogenesis of the neural stem cells (NSCs) from the adult brain and proves the superior effect compared to adult CSF.

The experiments of Alonso et al. (2017) and Madrigal et al. (2023) used a similar design, evaluating the stages of neural cell development (Beta3 tubulin, calretinin, NOD2), as well as cell proliferation (BrdU and SOX2) and migration (Doublecortin) of adult mouse brain explants in contact with latex microbeads soaked in different substances (basal medium and E-CSF, as well as adult CSF in the case of the study conducted by Alonso et al. (2017)). The differences between the two studies consist of the different areas of the brain that were used for the explants (subventricular zone for the first study, and subependymal zone, dentate migratory stream, and hilus for the second one) and the presence of the adult CSF as the comparator for the study of Alonso et al. (2017). For all brain areas, E-CSF beads, compared to basal medium beads, determined higher expression of markers for cell proliferation, for cell migration (in the rostral migratory stream, subependymal zone, dentate migratory stream, and hilus), and for cell neurodifferentiation (higher number of both young and differentiated neurons), except for young neurons in hilus explants.

Alonso et al. (2017) also demonstrated that adult CSF-soaked beads determine lower cell proliferation than E-CSF-soaked beads and lower neurogenetic effect (lower number of young neurons) than basal medium, with the majority of young neurons present expressing low level of Beta3 tubulin.

Madrigal et al. (2023) also compared the three different hippocampal zones. When young neurons were taken into consideration, basal medium-soaked beads did not determine any differences between zones, while E-CSF-soaked beads determined an increase from subependymal zone to dentate migratory stream to hilus. Conversely, when looking at differentiated neurons, the roles were reversed, E-CSF-soaked beads did not determine any difference, and basal medium-soaked beads showed the mentioned increase. Migration showed a specific pattern, similar between E-CSF and basal medium-soaked beads, with an increase of migratory markers from the dentate migratory stream to the subependymal zone to the hilus.

Precursor cells are still present in different areas of the adult brain. The key to neural regeneration seems to be the possible stimulation of these neuroprogenitors to differentiate in adult cells, through different chemical and biological factors that influence the neurogenic niche. The cited studies proved, by multiple designs, that E-CSF seems to be an excellent candidate for inducing these regenerative effects in the precursors present in the adult brain, with multiple possible applications in regenerative medicine.

### The Effects of Rat Embryonic CSF

#### Neural Cells—Embryonic Tissue

Martin et al. (2009) and Lamus et al. (2020) both used 12.5 days rat mesencephalic explants to test the effect of age-matched E-CSF compared to other culture conditions. Both studies used an in vivo control at 13.5 days gestational age and BrdU cells to investigate cell proliferation, and Beta3 tubulin for neurogenesis, respectively. Martin et al. (2009) showed that explants cultured in E-CSF determine better results (in terms of cell proliferation, survival, and neurogenesis) than the ones cultured in basal medium or fetal calf serum, but not as good as in vivo controls. These results are different from the ones Gato et al. (2005) found for chick E-CSF, that showed lower cell proliferation in comparison to fetal calf serum. In a similar manner, the study of Lamus et al. (2020) showed that adding E-CSF to the basal medium leads to better cell proliferation and neurogenesis than basal medium alone or supplemented with EGF, but comparing the results with those obtained by adding FGF2, E-CSF determines lower cell proliferation, and similar neurogenesis, with higher differentiation on the luminal surface induced by FGF2. Adding antagonists, such as anti-FGF2 antibodies and anti-EGF antibodies, diminishes the proliferative and neurogenetic effect of E-CSF, FGF2, and EGF.

The study of Miyan et al. (2020) investigated the effect of E-CSF soaked beads on rat brain slices, depending on the placement of the beads. They showed that when E-CSF only comes into contact with the pial surface of the brain, cell proliferation and migration around beads is minimal, similar to the placement of PBS-soaked beads. In contrast, when the beads are applied on the ventricular surface or on both surfaces, there is a significant increase in the proliferation and migration of neural cells. The study also concluded that when slices were cultured in E-CSF enriched medium there was higher proliferation and cell survival than when only PBS was added to the culture medium.

Yari et al. (2013) used neurospheres from embryonic rat subventricular zone as a substrate for different gestational ages E-CSF enriched medium. E-CSF from later development (E20) showed similar results to control, with lower cell proliferation and neurosphere size and differentiation oriented to glial cells, probably detrimental to neurons, compared to those determined by adding earlier E-CSF (E16,18).

#### Neural Cells—Neural Stem Cells

The study of Nabiuni et al. (2012) used PC12 cells (isolated from rat pheochromocytoma) as a model of neural progenitors. The authors were able to demonstrate that E-CSF was superior to control in terms of cell proliferation, for all gestational ages studied. Even though the cells cultured in 18 days E-CSF showed the highest amount of proliferation, this CSF did not determine any amount of differentiation (as opposed to all other ages E-CSF and basic fibroblast growth factor (bFGF) in which morphological differentiation or Beta3tubulin—neuronal marker, was present). MAP2 (neuronal marker) was not identified in cells cultured in basal medium or in E-CSF harvested at 18 days or 20 days GA, but present in the neurites for bFGF and 17 days and 19 days E-CSF cultured cells, and also in the cell bodies for cells cultured in 17 days E-CSF, that seems to have the highest neurogenetic effect. These results are aligned with the ones of Yari et al. (2013), suggesting that 20 days gestational age E-CSF is beginning to lose in part the neurogenetic effect and shift toward a more adult type CSF.

Peirouvi et al. (2015) used NSCs obtained from adult rat hippocampus and cultured them in a control medium, adult and embryonic CSF, for each condition analyzing the results for both high KCl and normal KCl levels. Their results showed that adult CSF determines lower, while embryonic CSF leads to higher proliferation and differentiation than control. They were also able to demonstrate that higher concentration of E-CSF in the medium and higher KCl level lead to higher proliferation. Another important result of the study is that the presence of adult CSF leads to a differentiation toward glial cells, while the presence of E-CSF leads to a differentiation toward neurons. For E-CSF cultured cells, the presence of higher KCl levels also leads to higher expression of neuronal markers.

### Mesenchymal Stem Cells

#### Neural Differentiation

All studies showed better neural differentiation when cells were treated with E-CSF than in negative control (that varied from basal medium to non-treated cells). In the study of Dorazehi et al. (2018), there was no evident difference in electronic microscopy between E-CSF treated and control cells, but the E-CSF group expressed neuronal markers that were absent in the control group. In terms of morphology, two other studies showed important differences between E-CSF treated and control: with neurites absent in basal medium cultured cells in the study of Mohammadi-Mahdiabadi-Hasani et al. (2020), and fibroblast morphology for non-treated cells and significant neurite length differences between E-CSF treated and non-treated cells in the study of Goudarzi et al. (2020). Goudarzi et al. (2020) also showed higher presence of young neurons in E-CSF treated than in control, and the presence of mature neurons only in E-CSF enriched cultures.

#### Cell Proliferation

Regarding cell proliferation, four of the studies showed a clear superiority of the cells treated with E-CSF (Dorazehi et al., 2018; Mohammadi-Mahdiabadi-Hasani et al., 2020; Shokohi et al., 2018a, 2018b), and the fifth demonstrated that human dental pulp stem cells proliferate in a time-dependent manner, with high proliferation at first, that progressively declines, the decrease being higher in the E-CSF group (Goudarzi et al., 2020). The time dependency is shown in the study published by Dorazehi et al. (2018) too, with proliferation increasing in the first 15 days, with a progressive decline until day 30. Two studies published by the same group (Shokohi et al., 2018a, 2018b) were able to show that higher concentrations of E-CSF (10%) lead to better proliferation that the lower concentrations (3 and 7%).

#### Comparison to Other Factors

Three of the studies used the addition of bFGF (Dorazehi et al., 2018; Shokohi et al., 2018a, 2018b), and one study (Mohammadi-Mahdiabadi-Hasani et al., 2020) the addition of Beta mercapto-ethanol to the culture medium, as a comparator for E-CSF (showing lower cell proliferation for beta mercapto-ethanol than basal medium). Comparing bFGF to E-CSF in terms of neurogenesis, the results were heterogeneous. While all studies showed that bFGF is superior to negative control, both studies of Shokohi et al. obtained higher neurite growth and neurogenic marker expression for bFGF-treated cells than E-CSF treated cells and Dorazehi et al. (2018) demonstrated higher neurogenic marker expression in E-CSF treated than in bFGF-treated cells. The concentration and GA of E-CSF in the studies were similar, thus the difference could not be explained by this aspect.

#### Different Gestational Ages

Three of the studies also compared different GA (from 17 days to 20 days and postnatal) of the E-CSF (Mohammadi-Mahdiabadi-Hasani et al., 2020; Shokohi et al., 2018a, 2018b). The studies published by the Shokohi et al. group showed that 17 days and 19 days GA E-CSF leads to better neurogenesis (neurite length and immunochemical markers) than 18 days E-CSF, and that 20 days gestational age E-CSF is similar to postnatal CSF, leading to lower differentiation than earlier E-CSF (19 days). Conversely, Mohammadi-Mahdiabadi-Hasani et al. (2020) obtained different results, showing no neurite growth on the cells cultured in 17 days E-CSF, and identified neurite growth in 18 days and 19 days E-CSF. One explanation for the identified differences might be the different stem cell types used.

#### Limitations

The current research on E-CSF presents several limitations that make it difficult to draw significant conclusions and to establish the next steps in translating the results in clinical practice. The most important one is the scarcity of studies on human E-CSF. We were able to identify only three such studies, each with small sample sizes, none of which investigated effect (Christiansen et al., 2000; Feliciano et al., 2014; Zappaterra et al., 2007). This is mainly because of the limited availability of human fetal tissue in general, due to the ethical concerns regarding its use, and to the additional technical difficulties of collecting E-CSF in the context of pregnancy termination, that often leads to blood contamination of the samples.

Another significant limitation is the variability in methodological approaches (species, gestational age, targeted substances—for identification, studied factors—for effect, analysis methods, etc.). The high heterogenicity makes it impossible to compare results of studies across different research groups, to conduct meta-analysis for quantitative results and to draw unitary conclusions.

The quality of the studies is also deficient, as it can be seen from our quality assessment through Nature Checklist (Cramond et al., 2016). Fundamental research does not benefit from extensive good practice guidelines as clinical research does. New instruments have emerged for better and unitary data reporting, such as the Nature Checklist (Cramond et al., 2016) and CAMARADE Checklist (Macleod et al., 2004), but they are still not widely used in the scientific community and a part of the included studies date before the publication of these guidelines.

#### Future Directions

The research of E-CSF has an important potential for translational science. In order to identify appropriate next steps in the domain, there is a need for a better description of the normal physiological pathways, using animal models and especially human samples. Even though the research community values novelty, setting a base of knowledge, through standardized methodologies and robust designs, is essential for the identification of relevant pathways in neurogenesis. Future studies should focus on several key areas to enhance our understanding of the role of E-CSF in neural development and disease:

Establishing biobanks for fetal tissues, with standardized collection protocols and informed consent, will provide valuable human samples for deeper insights into E-CSF molecular composition and function in development.

Investigating the effect of E-CSF not only on cell culture or explants, but on 3D models and neural organoids will help elucidate its role in brain development, offering a more physiologically relevant system to study early neural differentiation. Also, in vivo studies in adult animal models could be pursued to provide a comprehensive understanding of E-CSF impact on neuroregeneration.

There is a need for a better description of the E-CSF proteome, using the performant technologies available today, like liquid chromatography-mass spectrometry (LC–MS), for both untargeted and targeted approaches, offering reliable biomarkers for further research and potential therapeutic applications.

By advancing research in these areas, we can better understand E-CSF biological significance and its potential in neurodevelopmental disorders and regenerative medicine.

## Conclusion

The included studies showed that E-CSF is an important matrix with vast implications in neurodevelopment, with a specific composition (that varies between species and with gestational age), and not only nurturing, but also differentiating effects on neural progenitors.

All included studies showed better results in terms of proliferation, survival, and differentiation when cells were cultured in E-CSF, compared to basal medium.

We observed a decrease in the number of published studies in the last 5 years (the subject grew out of focus). Even though some studies focused on pathological models are still published, the normal composition and effects of E-CSF are not well enough known to draw significant conclusions.

Although many studies have been identified in our search, the quality of the results is relatively low, especially because of the available tissues and methods:

- Only four studies compared E-CSF with adult/postnatal CSF (only for effect and not in terms of composition),
- No studies used human E-CSF for the investigation of the effect on cell cultures and only three studies used human E-CSF for composition description,
- The widest range of identified substances was obtained using MS, but only three studies used mass spectrometry to assess the proteome,
- Only one study used human cells, and those were MSCs, (and a study used human amniotic membrane as a scaffold), no study used human NSCs,
- Some of the studies had contradictory results regarding the effect of different gestational ages E-CSF on cell cultures and the effect of E-CSF compared to other factors (bFGF, fetal serum) on cell cultures.

The studies used heterogenous protocols, with different E-CSF gestational age, concentration, comparators, method of E-CSF application (soaked beads, culture medium), different species cells, differences in cell cultures (species, cells vs explants), method for effect evaluation, that lead to a difficulty in drawing unitary conclusions. The heterogeneous nature of the study designs and experimental approaches showcase the need for standardized methodologies to better understand the unique properties and potential clinical applications of E-CSF.

Nevertheless, there is clear data supporting the effect of E-CSF in neural regeneration on animal models, through the proliferative and differentiating effects on progenitors. These studies create scientific support for further studies regarding these effects in human models. E-CSF neurogenetic effects are not only manifested in embryonic tissue, but also on progenitors from adult neural tissue, suggesting important clinical implications in regenerative medicine.

## Supplementary Information

Below is the link to the electronic supplementary material.

Supplementary file1 (XLSX 18 KB)

## Data Availability

No datasets were generated or analyzed during the current study.
